# Foot Muscle Size and Balance in Stroke: Evidence of Structural‐Functional Dissociation

**DOI:** 10.1002/jfa2.70206

**Published:** 2026-08-31

**Authors:** Xiaoya Zhang, Yaobin Wu, Guirong Liu

**Affiliations:** ^1^ Department of Rehabilitation Medicine The Third Affiliated Hospital of Sun Yat‐sen University Guangzhou China

**Keywords:** foot, hemiplegia, muscle, postural balance, skeletal, stroke, ultrasonography

## Abstract

**Purpose:**

This study investigated foot muscle and plantar fascia size measurements and their relationship with static balance dysfunction in stroke patients.

**Methods:**

This cross‐sectional study included 90 stroke patients (3–12 months post‐stroke) and 95 healthy controls. Ultrasound imaging quantified thickness and cross‐sectional area (CSA) of intrinsic foot muscles (e.g., flexor hallucis brevis), extrinsic foot muscles (e.g., tibialis anterior, peroneus longus), and plantar fascia segments. Force platform measured center‐of‐pressure (CoP) parameters during 30‐s static standing under eyes‐open and eyes‐closed conditions.

**Results:**

Stroke patients exhibited significantly reduced peroneus longus (*d* = 1.14, *p* < 0.001) and tibialis anterior (*d* = 0.70, *p* < 0.001) muscle sizes, reduced flexor hallucis brevis muscle thickness (*d* = 0.76, *p* < 0.001), and increased plantar fascia thickness in middle and posterior segments (*d* = 0.74, *p* < 0.001; *d* = 0.71, *p* < 0.001). In healthy controls, foot muscle parameters strongly correlated with balance metrics, with flexor hallucis brevis thickness showing significant correlation with mediolateral displacement under eyes‐closed conditions (*r* = −0.52, *p* < 0.001). However, no significant correlations between structural parameters and balance metrics were observed in stroke patients after false discovery rate correction, indicating structural‐functional dissociation.

**Conclusion:**

Although stroke patients exhibited mixed size changes in foot‐ankle structures, the dissociation between structural changes and balance function suggests that dysfunction is primarily attributable to central nervous system damage rather than to peripheral structural alterations. This implies that rehabilitation should focus on neuromuscular control training rather than on isolated muscle strengthening.

## 1 Introduction

Balance dysfunction is a major clinical manifestation in stroke patients, characterized by asymmetric static pressure distribution and increased elliptical sway area of the center‐of‐pressure (CoP) trajectory [1], along with compromised dynamic weight‐shifting capacity, shortened stride length, and prolonged double‐limb support phase during gait [2]. These functional impairments are closely associated with compromised neuromuscular coordination in foot‐ankle structures.

Under physiological conditions, foot‐ankle structures regulate arch configuration and joint stiffness to maintain stability [3, 4]. Post‐stroke patients frequently experience partial or complete loss of motor function in distal limbs due to central nervous system injury [5, 6, 7]. This is often followed by significant structural changes driven by multifactorial mechanisms including denervation, immobilization, and inflammation [8, 9], potentially impairing postural stability during standing.

Intervention targeting ankle‐foot structures, including stretching of intrinsic structures [10] and neuroadaptive training [11] have demonstrated improvements in balance function. However, it remains unclear whether structural remodeling or neuroadaptive impairment is the primary driver of post‐stroke balance dysfunction, creating clinical confusion. Therefore, measuring size of plantar structure may serve as a proxy for assessing the integrity of the distal sensorimotor subsystem, which underpins postural control. However, current compensatory approaches such as ankle‐foot orthoses [12, 13, 14] rely on functional metrics, leaving the underlying structure‐function relationships largely unexplored. Previous studies have examined structural and tissue‐level alterations in intrinsic foot muscles after stroke, but they did not explore whether these changes have functional relevance for balance. Calvo‐Lobo et al. (2018a) found that both hemiparetic and contralateral feet showed increased flexor hallucis brevis thickness and decreased middle plantar fascia thickness compared with healthy controls. A separate pixel analysis study by the same group (Calvo‐Lobo et al., 2018b) revealed reduced echogenicity in the abductor hallucis, suggestive of fatty infiltration or fibrosis. However, these studies were limited to intrinsic foot muscles and did not examine the relationship between structural changes and balance function. The present study addresses this gap by extending the scope to extrinsic foot muscles and directly testing the structure‐function relationship, defined here as the association between plantar structure size and static balance performance.

Testing this structure‐function relationship could elucidate structural remodeling’s contribution to dysfunction, providing evidence for developing stratified intervention, for example, by targeting central sensory integration versus peripheral muscle strengthening. To this end, ultrasound was selected to measure thickness and cross‐sectional area (CSA) of foot muscles and plantar fascia. Ultrasound effectively quantifies changes in muscle thickness and CSA, as well as structural remodeling, in limb musculature post‐stroke [8, 15, 16], and has demonstrated excellent reliability in elderly populations [17, 18, 19].

Force plate provides precise CoP parameters that align with balance assessment requirements. In post‐stroke patients, force plate testing more directly assesses the biomechanical role of foot‐ankle structures in maintaining dynamic balance than motion capture systems [20]. CoP parameters including displacement velocity and envelope area enable quantitative balance evaluation [21, 22].

This study primarily tests two hypotheses: (1) In healthy controls, the thickness of intrinsic and extrinsic foot muscles correlates with static balance performance, potentially reflecting their role in fine adjustment and proprioception; however, this structure‐function correlation is expected to be absent or altered in stroke patients due to impaired central integration. (2) Compared with healthy controls, stroke patients exhibit significant differences in the thickness of foot muscles and plantar fascia segments.

## 2 Methods

### 2.1 Study Design and Participants

This cross‐sectional study was conducted at the Third Affiliated Hospital of Sun Yat‐Sen University between June 2024 and June 2025. The study was approved by the institutional ethics committee (Approval No. RG2023‐077‐01, dated April 26, 2023) and conducted according to the Declaration of Helsinki. Participants were recruited from the inpatient and outpatient rehabilitation departments. Potentially eligible patients were identified through daily screening of hospital admission records and physician referrals. Research staff approached potential participants, provided verbal and written information about the study, and screened for initial eligibility. Those who met the preliminary criteria were scheduled for a full screening visit, during which written informed consent was obtained prior to any study procedures.

Stroke patients met the following criteria: (1) diagnosis confirmed by CT/MRI imaging [23, 24]; (2) age ≥ 18 years; (3) 3–12 months post‐stroke, covering the late subacute to early chronic phase [25]; (4) ability to stand independently for ≥ 30 seconds, which is the standard duration for quiet standing balance assessment in stroke populations [26]; (5) MMSE score ≥ 24 (elementary education) or ≥ 18 (no formal education), to ensure adequate comprehension of study instructions [27]; (6) mRS score 2‐4, indicating moderate to moderately severe disability with preserved capacity to participate in assessments [28]; (7) lower extremity Fugl‐Meyer score ≥ 20, ensuring sufficient lower extremity motor function for standing balance tasks [29]. Healthy controls were aged 18–80 years with gait speed ≥ 1.0 m/s, lower limb strength ≥ Grade 4, and no neurological disorders.

Exclusion criteria included foot deformities or surgery, uncontrolled hypertension, diabetic neuropathy, severe cardiopulmonary disease, vestibular dysfunction, recent botulinum toxin injection, pregnancy, and inability to follow protocols.

### 2.2 Sample Size Calculation

Sample size calculation was based on an effect sizes (Cohen’s *d* = 0.7) reported by previous studies examining fascial thickness differences in stroke patients [30]. Using G*Power 3.1 software with a two‐tailed independent *t*‐test (*α* = 0.05, 80% power), a minimum of 54 participants per group was required. To account for potential data collection challenges and enhance statistical robustness for subgroup analyses, the sample size was increased to 100 participants per group (100 stroke patients and 100 healthy controls). In the stroke group, participant dropout reasons were as follows: 3 participants were transferred to another medical institution, 2 due to transportation difficulties, and 5 due to cumbersome research procedures and poor compliance. In the healthy control group, 2 participants withdrew due to dissatisfaction with research procedures and 3 lost interest during the examinations.

### 2.3 Ultrasound Imaging

All ultrasound measurements were performed by an experienced physician with over 7 years of experience using a ultrasound device (uSmart3200T NexGen Ultrasound System, Terason, USA) equipped with a linear wideband array transducer (model: 15L4A Linear Array, frequency range: 4–15 MHz). The evaluator was blinded to group allocation during assessment.

Size measurements (thickness and CSA) of intrinsic foot muscles, extrinsic foot muscles, and plantar fascia were measured following the standardized protocol established by Crofts et al. [31]. The measured structures included: (1) plantar fascia: anterior, middle, and posterior segments; (2) extrinsic muscles: tibialis anterior (TA), peroneus longus (PL), flexor digitorum longus (FDL), and flexor hallucis longus (FHL); (3) intrinsic foot muscles: flexor digitorum brevis (FDB), flexor hallucis brevis (FHB), and abductor hallucis (AbH). The selected intrinsic foot muscles (FHB, FDB, AbH) are key contributors to medial longitudinal arch support and fine postural control. The extrinsic muscles TA and PL were chosen for their roles in ankle dorsiflexion and eversion, respectively‐functions frequently impaired post‐stroke and critical for upright stance. FDL and FHL were included as they act on the toe flexors, contributing to forefoot stability. In addition, plantar fascia thickness was measured because it serves as a passive stabilizer of the foot arch and has been reported to undergo structural changes following stroke [30]. Together, these structures represent the primary soft‐tissue components of the distal sensorimotor subsystem involved in static balance control. CSA was obtained only for muscles with clear transverse‐plane borders (AbH, PL, TA). For FDB, FDL, and FHL—which are smaller and anatomically more challenging—only thickness was measured. This decision was based on the higher variability of CSA reported for these structures in the Crofts et al. protocol (e.g., FDL CSA Limit of agreement (LoA) = 26%), whereas thickness demonstrated consistently excellent reliability (ICC 0.90–0.97).

Participants were positioned prone for scanning the plantar fascia, FHB, and FDB, and supine for scanning the AbH, FDL, FHL, TA, and PL. The plantar fascia was imaged longitudinally along the line from the medial calcaneal tubercle to the second toe at three sites: calcaneal insertion, navicular tubercle, and second metatarsal head, with the toes maintained in a relaxed resting position to standardize fascial tension. For intrinsic foot muscles, FHB thickness was measured longitudinally along the first metatarsal shaft at the level of the navicular tuberosity, targeting the medial head at its thickest portion—thus standardizing the two‐headed nature of the muscle; FDB was imaged along the line from the medial calcaneal tubercle to the third toe; and AbH was scanned between the medial calcaneal tuberosity and the navicular tuberosity. For extrinsic muscles, FDL and FHL CSA were acquired at 50% of the distance between the medial tibial plateau and the inferior border of the medial malleolus; PL CSA at 50% between the fibular head and the inferior border of the lateral malleolus; and TA thickness at 20% of this distance, with the ankle maintained in neutral using a foam support. To ensure reproducibility, two independent images were acquired at each measurement site, with the probe removed and repositioned between acquisitions; the mean of the two measurements was used for analysis. All offline measurements were performed by a single assessor blinded to group allocation using ImageJ software (National Institutes of Health, Bethesda, MD, USA). Images were calibrated from pixels to centimeters using the straight‐line tool. Muscle thickness was defined as the linear distance between the superficial and deep fascial borders at the thickest portion of the muscle, measured with the straight‐line tool. CSA was obtained by manually tracing the inner contour of the muscle fascial border using the polygon tool. For plantar fascia thickness, the perpendicular distance between its superficial and deep margins was measured at each of the three predefined sites.

### 2.4 Static Balance Testing

Static balance was assessed using a force platform (Medtrack‐Gait 0.5M, Zhizu Network Technology Co. Ltd., China) sampling at 1000 Hz. AiFoot software (sampling frequency 200 Hz) was used to record center of pressure (CoP) trajectory parameters. Anteroposterior (AP) displacement was defined as the range of CoP movement in the AP direction; mediolateral (ML) displacement as the range of CoP movement in the ML direction; and envelope area (EA) as the area of the 95% confidence ellipse of the CoP trajectory. The selected CoP parameters (AP displacement, ML displacement, and envelope area) have been reported to have acceptable test‐retest reliability in stroke populations (ICC range 0.71–0.91) [32].

Participants performed 30‐s static double‐leg standing tests under both eyes‐open (EO) and eyes‐closed (EC) conditions, with three repetitions per condition, in a randomized order across participants. All tests were conducted barefoot. During testing, participants maintained a shoulder‐width stance with hands naturally positioned at their sides and feet placed at a consistent position marked on the force platform. During EO trials, participants focused on a cross mark placed at eye level 5 m ahead. The software initiated recording of CoP trajectory after a 5‐s countdown. For EC trials, participants closed their eyes after the software countdown was completed. Adequate rest intervals (at least 30 seconds) were provided between consecutive trials to minimize fatigue effects. CoP signals were filtered offline using a 4th‐order low‐pass Butterworth filter with a 10 Hz cutoff frequency. Trials with visible artifacts (e.g., coughing, limb movement, or loss of balance) were excluded and repeated after a rest period. The mean of the three repetitions was used for analysis.

### 2.5 Outcome Measures

The primary outcome measure was the correlation between foot structure size measurements and static standing balance, assessed by: (1) size measurements (thickness and CSA) of the plantar fascia (anterior, middle, posterior segments), extrinsic foot muscle (TA, PL, FDL, FHL), and intrinsic foot muscle (FDB, FHB, AbH); and (2) static standing balance parameters including AP and ML displacement, and EA of CoP under EO and EC conditions.

The secondary outcome measure was the group difference in size measurements (thickness and CSA) of the plantar fascia, extrinsic foot muscles, and intrinsic foot muscles between stroke and control groups.

### 2.6 Statistical Analysis

Statistical analysis was performed using SPSS version 27.0 (IBM Corp., Armonk, NY, USA). The normality of continuous variables was examined using the Shapiro‐Wilk test. Normally distributed variables were expressed as mean ± standard deviation, whereas non‐normally distributed variables were expressed as median (interquartile range).

For group comparisons, independent t‐tests were used for normally distributed data, and Mann‐Whitney U tests were applied for non‐normally distributed data. ANCOVA was performed with sex as a covariate when baseline differences in sex distribution between the two groups were statistically significant. Hierarchical regression modeling was performed separately for stroke and healthy groups to examine structure‐function relationships, adjusting for sex and age. To assess potential multicollinearity among predictors, variance inflation factors (VIF) were calculated for all variables in the regression models; VIF values below 2.5 were considered indicative of no significant multicollinearity.

Subgroup analyses compared different stroke types (ischemic vs. hemorrhagic), disease stages (early stage 3–6 months vs. late stage 7–12 months), and hemiplegic sides (left vs. right). False discovery rate (FDR) correction using the Benjamini‐Hochberg method was applied to regression analyses. Effect sizes were interpreted using Cohen’s conventions, with |β| or |r| values < 0.3 considered weak, 0.3–0.5 moderate, and > 0.5 strong. All collected CoP data were retained for analysis. No outliers were excluded. Statistical significance was set at *p* < 0.05.

## 3 Results

### 3.1 Participant Characteristics

185 participants were analyzed (90 stroke patients, 95 healthy controls). The baseline characteristics of the study participants are summarized in Table 1. There was a significant difference in sex distribution between groups, with more males in the stroke group (74.4% vs. 47.4%, *p* = 0.001). No significant differences were observed in age, height, weight, or BMI between groups (all *p* > 0.05).

**TABLE 1:** Baseline characteristics of study participants.

Characteristic	Stroke group (*n* = 90)	Control group (*n* = 95)	*p* value	Effect size
Demographics				
Sex (male/female)	67/23	45/50	0.001	*φ* = 0.28
Age (years)	58.4 ± 9.2	56.8 ± 11.4	0.26	*d* = 0.15
Height (cm)	167.2 ± 7.8	165.4 ± 8.2	0.12	*d* = 0.22
Weight (kg)	68.9 ± 12.1	66.2 ± 10.8	0.09	*d* = 0.24
BMI (kg/m^2^)	24.6 ± 3.4	24.1 ± 3.1	0.28	*d* = 0.15
Clinical characteristics				
Time since stroke (months)	6.8 ± 2.9	—	—	—
Stroke type		—		
‐ Ischemic	72 (80.0%)	—	—	—
‐ Hemorrhagic	18 (20.0%)	—	—	—
Hemiplegic side		—		
‐ Left	43 (47.8%)	—	—	—
‐ Right	47 (52.2%)	—	—	—
Functional assessments				
MMSE score	26.8 ± 2.4	28.9 ± 1.6	< 0.001	*d* = 1.02
Berg balance scale	48.2 ± 6.8	55.7 ± 1.8	< 0.001	*d* = 1.47
Modified rankin scale	2.6 ± 0.7	0.1 ± 0.3	< 0.001	*d* = 4.89
Fugl‐Meyer lower extremity	28.4 ± 4.2	34.0 ± 0.0	< 0.001	*d* = 2.14
Gait speed (m/s)	0.89 ± 0.18	1.23 ± 0.15	< 0.001	*d* = 2.07

*Note:* Data are presented as mean ± standard deviation or number (percentage).

Abbreviations: BMI: body mass index; MMSE: Mini‐Mental State Examination.

Among stroke patients, the mean time since stroke onset was 6.8 ± 2.9 months, with 80.0% having ischemic stroke and 20.0% having hemorrhagic stroke. The distribution of hemiplegic sides was relatively balanced, with 47.8% having left‐sided hemiplegia and 52.2% having right‐sided hemiplegia. As expected, stroke patients demonstrated significantly lower functional assessment scores compared with controls, including MMSE (26.8 ± 2.4 vs. 28.9 ± 1.6, *p* < 0.001), Berg Balance Scale (48.2 ± 6.8 vs. 55.7 ± 1.8, *p* < 0.001), and gait speed (0.89 ± 0.18 vs. 1.23 ± 0.15 m/s, *p* < 0.001).

### 3.2 Foot Structure Size Parameters

Ultrasound assessment of foot structure size parameters revealed significant differences between stroke patients and healthy controls (Table 2). Regarding plantar fascia thickness, stroke patients showed significantly increased thickness in the middle segment (4.8 ± 0.9 vs. 4.2 ± 0.7 mm, *p* < 0.001, *d* = 0.74) and posterior segment (5.1 ± 1.1 vs. 4.4 ± 0.8 mm, *p* < 0.001, *d* = 0.71), whereas the anterior segment showed no significant difference (*p* = 0.28).

**TABLE 2:** Foot structure morphological parameters between groups.

Structure category	Parameter	Stroke group	Control group	Difference (95% CI)	*p* value	FDR q‐value	Effect size
Plantar fascia							
Anterior	Thickness (mm)	3.2 ± 0.6	3.1 ± 0.4	0.1 [−0.1, 0.3]	0.28	0.42	*d* = 0.19
Middle	Thickness (mm)	4.8 ± 0.9	4.2 ± 0.7	0.6 [0.3, 0.9]	< 0.001	0.003	*d* = 0.74
Posterior	Thickness (mm)	5.1 ± 1.1	4.4 ± 0.8	0.7 [0.4, 1.0]	< 0.001	0.002	*d* = 0.71
Intrinsic foot muscles							
Flexor hallucis brevis	Thickness (mm)	8.9 ± 1.8	10.2 ± 1.6	−1.3 [−1.8, −0.8]	< 0.001	0.001	*d* = 0.76
Flexor hallucis brevis	CSA (cm^2^)	1.24 ± 0.31	1.18 ± 0.24	0.06 [−0.03, 0.15]	0.18	0.31	*d* = 0.22
Flexor digitorum brevis	Thickness (mm)	9.8 ± 2.1	10.4 ± 1.9	−0.6 [−1.2, 0.0]	0.048	0.096	*d* = 0.30
Abductor hallucis	Thickness (mm)	7.6 ± 1.4	7.8 ± 1.2	−0.2 [−0.6, 0.2]	0.32	0.48	*d* = 0.15
Abductor hallucis	CSA (cm^2^)	2.18 ± 0.52	1.89 ± 0.41	0.29 [0.14, 0.44]	< 0.001	0.002	*d* = 0.62
Extrinsic foot muscles							
Peroneus longus	Thickness (mm)	12.8 ± 2.9	16.2 ± 3.1	−3.4 [−4.3, −2.5]	< 0.001	< 0.001	*d* = 1.14
Peroneus longus	CSA (cm^2^)	3.42 ± 0.89	4.18 ± 0.92	−0.76 [−1.02, −0.50]	< 0.001	< 0.001	*d* = 0.84
Tibialis anterior	Thickness (mm)	18.9 ± 4.2	21.7 ± 3.8	−2.8 [−4.0, −1.6]	< 0.001	0.001	*d* = 0.70
Tibialis anterior	CSA (cm^2^)	5.24 ± 1.32	6.01 ± 1.18	−0.77 [−1.13, −0.41]	< 0.001	0.002	*d* = 0.62
Flexor digitorum longus	Thickness (mm)	11.4 ± 2.6	12.1 ± 2.4	−0.7 [−1.4, 0.0]	0.052	0.104	*d* = 0.28
Flexor hallucis longus	Thickness (mm)	13.2 ± 3.1	13.8 ± 2.7	−0.6 [−1.4, 0.2]	0.15	0.26	*d* = 0.21

*Note:* Data are presented as mean ± standard deviation.

Abbreviations: CI: confidence interval; CSA: cross‐sectional area; FDR: false discovery rate.

For intrinsic foot muscles, stroke patients demonstrated significantly reduced flexor hallucis brevis thickness compared with controls (8.9 ± 1.8 vs. 10.2 ± 1.6 mm, *p* < 0.001, *d* = 0.76), whereas the CSA showed no significant difference (*p* = 0.18). Flexor digitorum brevis thickness was marginally reduced in stroke patients (9.8 ± 2.1 vs. 10.4 ± 1.9 mm, *p* = 0.048), but this did not survive FDR correction (*q* = 0.096). Conversely, CSA of abductor hallucis was significantly increased in stroke patients (2.18 ± 0.52 vs. 1.89 ± 0.41 cm^2^, *p* < 0.001, *d* = 0.62), whereas thickness remained unchanged (*p* = 0.32).

**FIGURE 1:**
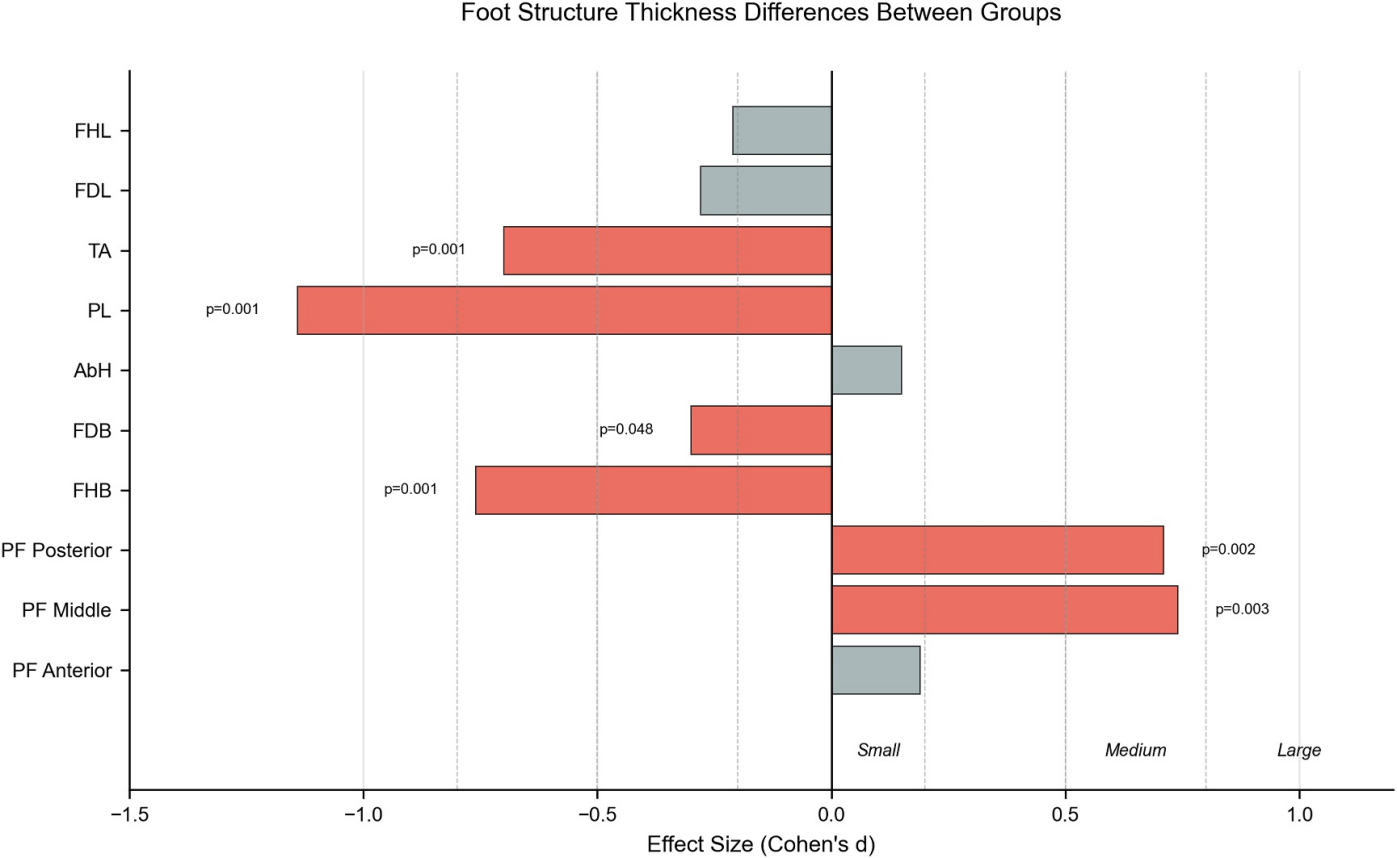
Effect sizes for foot structure differences between groups. Cohen's d values showing magnitude of difference between stroke patients and controls. Negative values (red) indicate reduced thickness; positive values (gray) indicate increased thickness in stroke patients.

Among extrinsic foot muscles, stroke patients exhibited significant smaller peroneus longus and tibialis anterior muscle sizes. Peroneus longus thickness was markedly reduced (12.8 ± 2.9 vs. 16.2 ± 3.1 mm, *p* < 0.001, *d* = 1.14), representing the largest effect size observed in the study. Similarly, tibialis anterior thickness was significantly decreased (18.9 ± 4.2 vs. 21.7 ± 3.8 mm, *p* < 0.001, *d* = 0.70). The CSAs of both muscles showed corresponding reductions (*p* < 0.001 for both). No significant differences were observed in flexor digitorum longus or flexor hallucis longus thickness between groups (*p* > 0.05). The effect sizes and percentage changes in muscle structural parameters are shown in (Figure 1) and (Supporting Information S1: Figure S1).

### 3.3 Static Balance Function Parameters

Stroke patients demonstrated significantly impaired static balance function compared with healthy controls across all measured parameters (Table 3). For center of pressure mediolateral displacement, stroke patients showed greater sway in both eyes‐open (12.8 ± 4.2 vs. 6.9 ± 2.1 mm, *p* < 0.001, *d* = 1.73) and eyes‐closed conditions (16.4 ± 5.8 vs. 9.2 ± 3.1 mm, *p* < 0.001, *d* = 1.51). Similarly, anteroposterior displacement was significantly increased in stroke patients under both visual conditions (eyes‐open: 18.7 ± 6.1 vs. 11.3 ± 3.4 mm, *p* < 0.001, *d* = 1.44; eyes‐closed: 22.9 ± 7.8 vs. 14.6 ± 4.2 mm, *p* < 0.001, *d* = 1.28).

**TABLE 3:** Static balance function parameters between groups.

Balance parameter	Test condition	Stroke group	Control group	Difference (95% CI)	*p* value	FDR q‐value	Effect size
CoP mediolateral displacement (mm)							
	Eyes open	12.8 ± 4.2	6.9 ± 2.1	5.9 [4.8, 7.0]	< 0.001	< 0.001	*d* = 1.73
	Eyes closed	16.4 ± 5.8	9.2 ± 3.1	7.2 [5.8, 8.6]	< 0.001	< 0.001	*d* = 1.51
CoP anteroposterior displacement (mm)							
	Eyes open	18.7 ± 6.1	11.3 ± 3.4	7.4 [5.9, 8.9]	< 0.001	< 0.001	*d* = 1.44
	Eyes closed	22.9 ± 7.8	14.6 ± 4.2	8.3 [6.5, 10.1]	< 0.001	< 0.001	*d* = 1.28
CoP envelope area (mm^2^)							
	Eyes open	286.4 ± 142.8	98.7 ± 41.2	187.7 [157.1, 218.3]	< 0.001	< 0.001	*d* = 1.71
	Eyes closed	412.6 ± 198.5	156.3 ± 67.4	256.3 [214.7, 297.9]	< 0.001	< 0.001	*d* = 1.62

*Note:* Data are presented as mean ± standard deviation.

Abbreviations: CI: confidence interval; CoP: center of pressure; FDR: false discovery rate.

**FIGURE 2:**
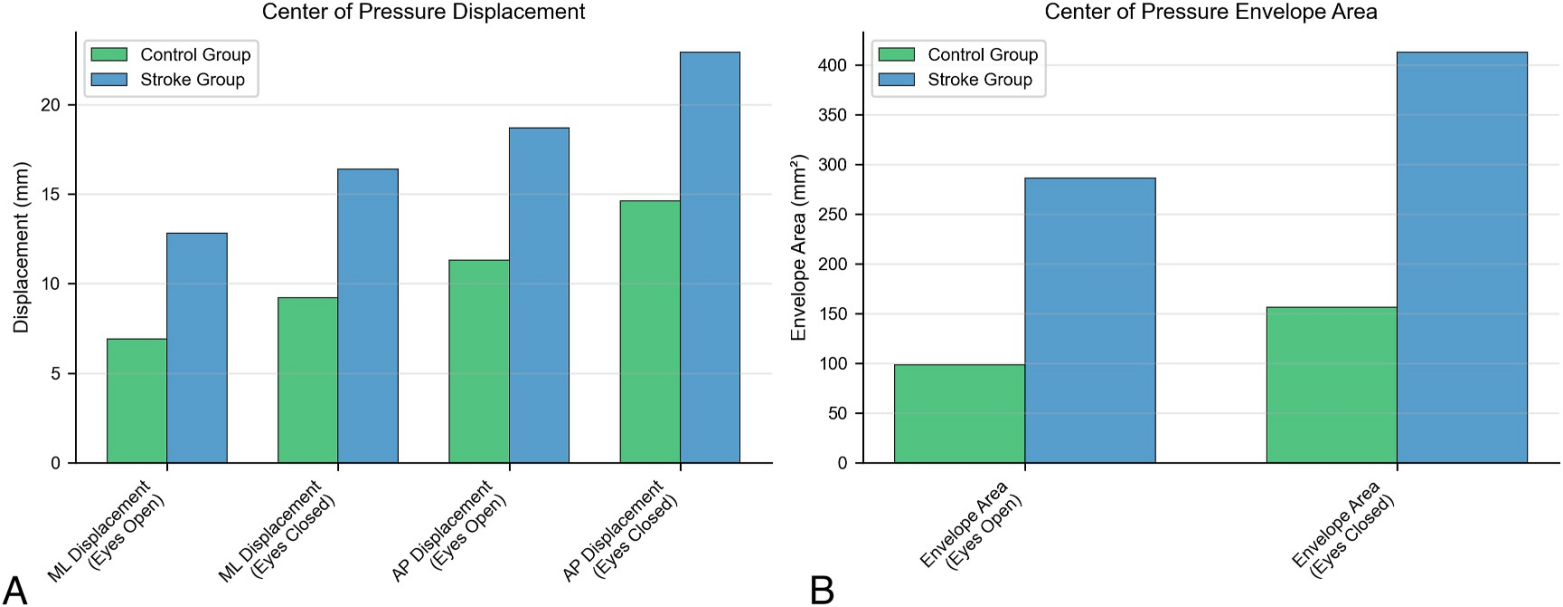
Center of pressure parameters comparison between groups. (A) Mediolateral and anteroposterior displacement in millimeters. (B) Envelope area in square millimeters. Stroke patients (blue) show significantly greater values than controls (green) in both visual conditions.

The center of pressure envelope area, representing the overall postural stability, was markedly larger in stroke patients compared with controls. Under eyes‐open conditions, stroke patients exhibited an envelope area of 286.4 ± 142.8 mm^2^ compared with 98.7 ± 41.2 mm^2^ in controls (*p* < 0.001, *d* = 1.71). This difference was even more pronounced under eyes‐closed conditions (412.6 ± 198.5 vs. 156.3 ± 67.4 mm^2^, *p* < 0.001, *d* = 1.62). All balance parameters showed large effect sizes (Figure 2).

### 3.4 Structure‐Function Association Analysis

The association analysis between foot structure thickness and static balance parameters revealed contrasting patterns between healthy controls and stroke patients (Table 4). In healthy controls, several significant associations were observed after FDR correction. Flexor hallucis brevis thickness showed strong negative associations with mediolateral displacement under eyes‐closed conditions (*β* = −0.52, *p* < 0.001, *q* = 0.002) and envelope area under eyes‐closed conditions (*β* = −0.48, *p* = 0.001, *q* = 0.008). CSA of abductor hallucis was significantly associated with anteroposterior displacement under both eyes‐open (*β* = −0.43, *p* = 0.003, *q* = 0.018) and eyes‐closed conditions (*β* = −0.39, *p* = 0.009, *q* = 0.041). Additionally, peroneus longus thickness showed a significant negative association with mediolateral displacement under eyes‐open conditions (*β* = −0.41, *p* = 0.005, *q* = 0.025), and CSA of tibialis anterior associated with envelope area under eyes‐open conditions (*β* = −0.37, *p* = 0.012, *q* = 0.048).

**TABLE 4:** Structure‐function association analysis.

		Healthy controls	Stroke patients
Structure	Balance parameter	β coefficient [95% CI] |*p* value/FDR q‐value	β coefficient [95% CI] |*p* value/FDR q‐value
Flexor hallucis brevis thickness			
	CoP mediolateral displacement (eyes closed)	−0.52 [−0.78, −0.26] | < 0.001/0.002	−0.12 [−0.38, 0.14] | 0.36/0.68
	CoP envelope area (eyes closed)	−0.48 [−0.74, −0.22] | 0.001/0.008	−0.09 [−0.35, 0.17] | 0.49/0.76
Abductor hallucis CSA			
	CoP anteroposterior displacement (eyes open)	−0.43 [−0.71, −0.15] | 0.003/0.018	−0.18 [−0.44, 0.08] | 0.17/0.42
	CoP anteroposterior displacement (eyes closed)	−0.39 [−0.68, −0.10] | 0.009/0.041	−0.15 [−0.41, 0.11] | 0.25/0.54
Peroneus longus thickness			
	CoP mediolateral displacement (eyes open)	−0.41 [−0.69, −0.13] | 0.005/0.025	−0.21 [−0.47, 0.05] | 0.11/0.31
Tibialis anterior CSA			
	CoP envelope area (eyes open)	−0.37 [−0.66, −0.08] | 0.012/0.048	−0.24 [−0.50, 0.02] | 0.068/0.22
Plantar fascia posterior			
	CoP mediolateral displacement (eyes closed)	−0.16 [−0.44, 0.12] | 0.26/0.52	0.28 [0.02, 0.54] | 0.034/0.16
Flexor digitorum brevis thickness			
	CoP anteroposterior displacement (eyes open)	−0.22 [−0.50, 0.06] | 0.13/0.31	−0.26 [−0.52, 0.00] | 0.049/0.18

*Note:* Analysis adjusted for sex and age. Only associations with *p*< 0.05 are shown.

Abbreviations: CI: confidence interval; CoP: center of pressure; CSA: cross‐sectional area; FDR: false discovery rate.

**FIGURE 3:**
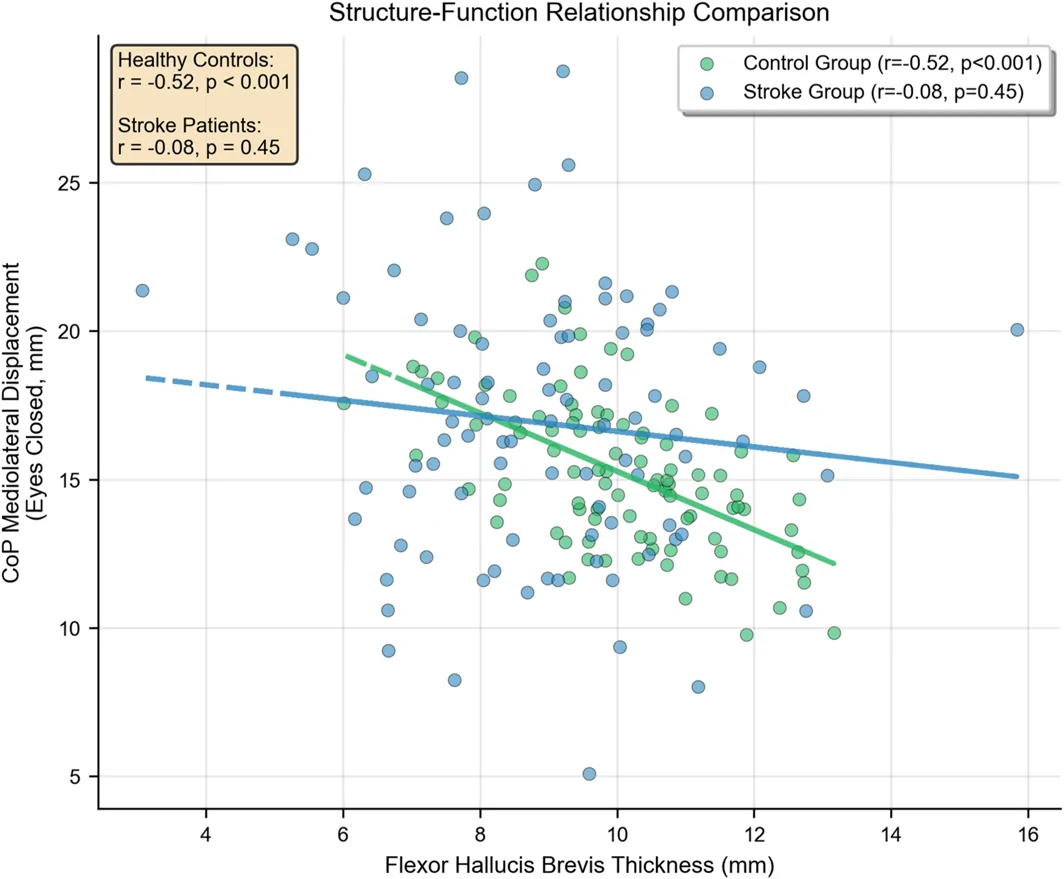
Structure‐function association between FHB thickness and CoP displacement. Scatter plot showing unadjusted linear relationship (simple linear regression) in healthy controls and stroke patients.

In stark contrast, stroke patients showed no significant associations between any foot structure parameters and balance metrics after FDR correction. Although some weak associations were observed with uncorrected *p* values, including plantar fascia posterior segment with mediolateral displacement (*β* = 0.28, *p* = 0.034, *q* = 0.16) and flexor digitorum brevis thickness with anteroposterior displacement (*β* = −0.26, *p* = 0.049, *q* = 0.18), none survived multiple comparison correction. The unadjusted linear relationship between FHB thickness and CoP displacement is illustrated in Figure 3.

### 3.5 Subgroup Analysis

Subgroup analyses explored potential differences based on stroke characteristics (Table 5). Comparison between ischemic and hemorrhagic stroke patients revealed no significant differences in foot structure thickness or balance function parameters (all *p* > 0.05). The effect sizes were small to moderate, suggesting that stroke etiology does not substantially influence foot structural changes or balance dysfunction patterns.

**TABLE 5:** Subgroup analysis results.

Analysis type	Parameter	Group 1	Group 2	*p* value	Effect size
Stroke type comparison		Ischemic stroke (*n* = 72)	Hemorrhagic stroke (*n* = 18)		
	Peroneus longus thickness (mm)	12.6 ± 2.8	13.6 ± 3.2	0.18	*d* = 0.33
	Tibialis anterior thickness (mm)	18.7 ± 4.1	19.8 ± 4.5	0.31	*d* = 0.26
	Flexor hallucis brevis thickness (mm)	8.8 ± 1.7	9.4 ± 2.1	0.22	*d* = 0.32
	CoP mediolateral displacement (eyes closed, mm)	16.7 ± 5.9	15.3 ± 5.4	0.34	*d* = 0.25
	CoP anteroposterior displacement (eyes closed, mm)	23.1 ± 7.9	22.2 ± 7.5	0.66	*d* = 0.12
Disease stage comparison		Early stage (3–6 months, *n* = 48)	Late stage (7–12 months, *n* = 42)		
	CoP mediolateral displacement (eyes closed, mm)	17.2 ± 6.1	15.4 ± 5.3	0.13	*d* = 0.31
	CoP anteroposterior displacement (eyes closed, mm)	24.1 ± 8.2	21.4 ± 7.2	0.09	*d* = 0.35
	CoP envelope area (eyes closed, mm^2^)	441.8 ± 215.6	378.2 ± 173.4	0.11	*d* = 0.33
	Peroneus longus thickness (mm)	12.4 ± 2.7	13.3 ± 3.1	0.15	*d* = 0.31
	Flexor hallucis brevis thickness (mm)	8.7 ± 1.9	9.2 ± 1.7	0.19	*d* = 0.28
Hemiplegic side comparison		Left hemiplegia (*n* = 43)	Right hemiplegia (*n* = 47)		
	CoP mediolateral displacement (eyes closed, mm)	16.8 ± 5.6	16.0 ± 5.9	0.51	*d* = 0.14
	CoP anteroposterior displacement (eyes closed, mm)	23.4 ± 7.5	22.4 ± 8.1	0.54	*d* = 0.13
	Peroneus longus thickness (mm)	12.5 ± 2.8	13.1 ± 3.0	0.34	*d* = 0.21

*Note:* Data are presented as mean ± standard deviation.

Abbreviation: CoP: center of pressure.

**FIGURE 4:**
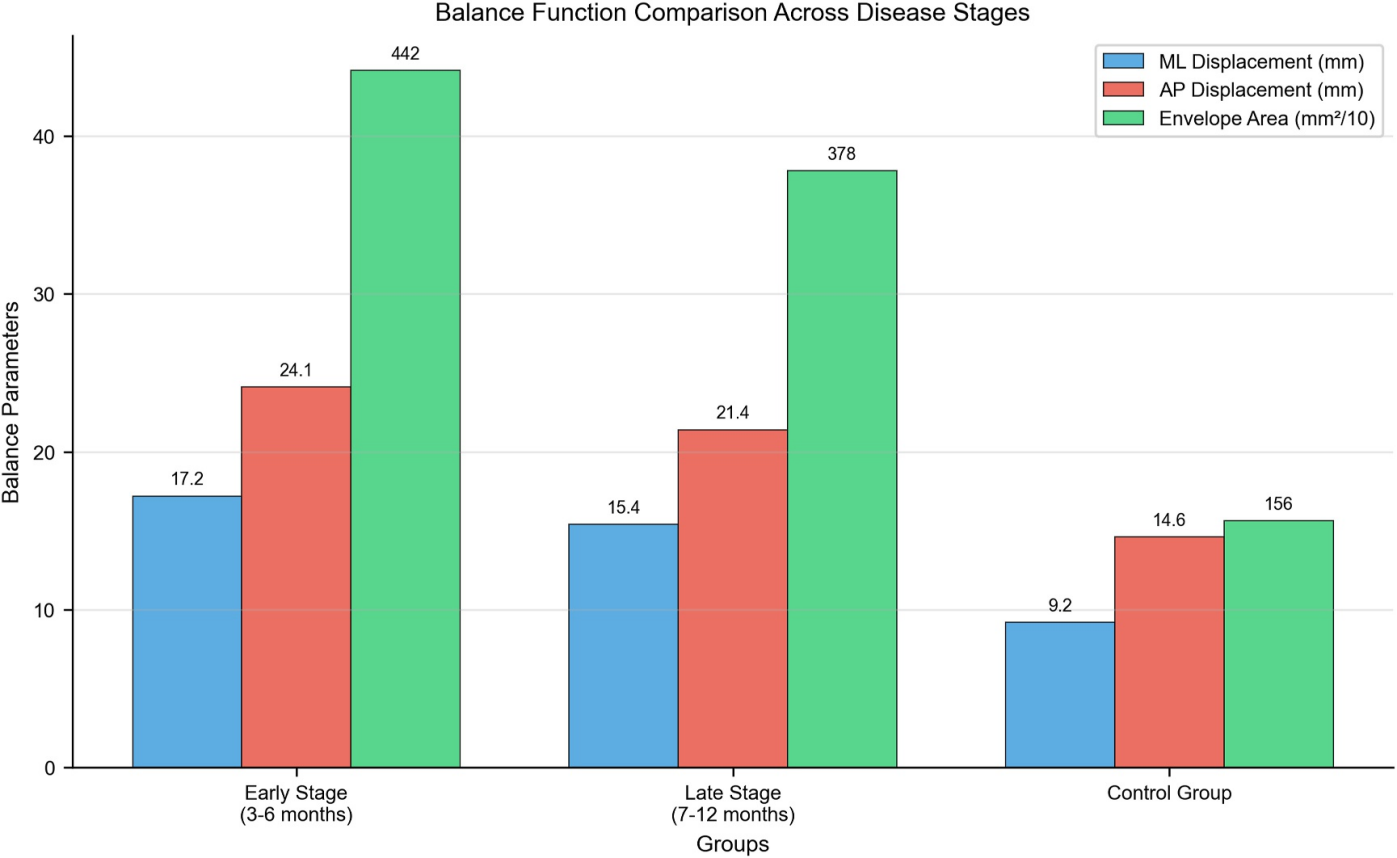
Comparison of static balance parameters across disease stages and healthy controls. Bars represent mediolateral displacement (ML, blue), anteroposterior displacement (AP, red), and envelope area (EA, green, mm^2^/10) during 30‐second static standing tests with eyes closed.

Disease stage analysis comparing early‐stage (3–6 months) and late‐stage (7–12 months) stroke patients showed no significant differences in balance function parameters. Early‐stage patients demonstrated numerically higher values for mediolateral displacement (17.2 ± 6.1 vs. 15.4 ± 5.3 mm, *p* = 0.13), anteroposterior displacement (24.1 ± 8.2 vs. 21.4 ± 7.2 mm, *p* = 0.09), and envelope area (441.8 ± 215.6 vs. 378.2 ± 173.4 mm^2^, *p* = 0.11), but these differences did not reach statistical significance. Similarly, no significant differences were observed in foot structure thickness between disease stages (Figure 4).

Comparison between left and right hemiplegic sides revealed no significant differences in any measured parameters (all *p* > 0.05), suggesting that the side of brain lesion does not differentially affect foot structure or balance function in this cohort.

### 3.6 Multivariable Regression Analysis

A multivariable regression model was constructed for healthy controls to identify the best predictors of mediolateral displacement under eyes‐closed conditions (Table 6). The final model included flexor hallucis brevis thickness, peroneus longus thickness, age, and sex as predictor variables. The model explained 38% of the variance in balance performance (*R* ^2^ = 0.38, *p* < 0.001). Flexor hallucis brevis thickness emerged as the strongest predictor (standardized *β* = −0.42, *p* < 0.001), followed by peroneus longus thickness (standardized *β* = −0.29, *p* = 0.009). Age and sex were not significant predictors in the model (*p* > 0.05). The variance inflation factors for all variables were below 1.2, indicating no multicollinearity issues.

**TABLE 6:** Multivariable regression model for healthy controls (dependent variable: CoP mediolateral displacement eyes closed).

Predictor variable	β coefficient	Standard error	Standardized *β*	t value	*p* value	VIF
Constant	18.42	2.86	—	6.44	< 0.001	—
Flexor hallucis brevis thickness	−0.68	0.18	−0.42	−3.78	< 0.001	1.12
Peroneus longus thickness	−0.24	0.09	−0.29	−2.67	0.009	1.08
Age	0.02	0.03	0.08	0.67	0.51	1.15
Sex (female)	0.81	0.52	0.17	1.56	0.12	1.09

*Note:* Model *R* ^2^ = 0.38, Adjusted *R* ^2^ = 0.35, F(4,90) = 13.8.

Abbreviation: VIF: variance inflation factor.

## 4 Discussion

This study revealed selective structural differences in foot musculature and plantar fascia among stroke patients and marked balance dysfunction. Most importantly, we identified a structural‐functional dissociation phenomenon where size of foot muscle no longer predicts balance performance in stroke patients, despite preserved structure‐function correlations in healthy controls.

This dissociation may first be explained by the nature of the assessment task itself. Static standing primarily relies on passive structural stiffness of the foot supported by plantar fascia and osseoligamentous structures [4], as well as spinal reflex‐mediated anti‐gravity muscle tone [33]. This is because intrinsic foot muscles provide minimally to active force generation during such low‐load tasks.

Additionally, this dissociation was evident even under eyes‐open conditions, which may reflect a broader impairment in the central reweighting and integration of multiple sensory inputs—including visual, proprioceptive, and vestibular information—for postural control, as previously described in stroke populations [34, 35]. The finding that CoP displacement increased further when vision was removed (Table 3) is consistent with this interpretation: without visual feedback, the central nervous system may be unable to adequately reweight remaining proprioceptive and vestibular inputs, further exposing the underlying integration deficit.

Furthermore, static balance relies on a hierarchical neural architecture. At the spinal level, short‐latency stretch reflexes provide rapid, automatic postural responses that can be selectively facilitated by postural instability [36]—a mechanism essential for maintaining basic muscle tone. However, supraspinal modulation is also required to adapt to varying environmental demands. Cortical pathways contribute to predictive and feedback control, enabling flexible, context‐dependent adjustments, whereas brainstem pathways support gross postural stability and locomotion [37]. Stroke disrupts these supraspinal inputs, impairing the ability to modulate postural responses according to task demands [38]. Although preserved spinal circuits may maintain basic tone, the loss of supraspinal drive likely contributes to progressive muscle size reduction in the absence of adequate neural input.

The general dissociation described above is further illustrated by the specific behavior of the peroneus longus. Among extrinsic muscles, the peroneus longus demonstrated the largest effect size of reduction (*d* = 1.14), which aligns with its critical role in dynamic balance control. Electrical stimulation of this muscle can effectively improve mediolateral dynamic balance, but has no significant effect on static balance [39].This dissociation between dynamic and static effects directly supports the notion that PL size reduction does not primarily explain static balance impairment in stroke patients.

The pronounced PL reduction likely reflects its high vulnerability to post‐stroke neural and mechanical changes. Approximately 80% of stroke patients exhibit abnormal gait with ankle and foot spasticity, predominantly involving plantarflexor and invertor muscle groups [40]. The PL, with its distal insertion into the first metatarsal and medial cuneiform, predominantly functions in foot pronation and abduction and must counteract these spastic forces to maintain dynamic joint stability [41], making its neural control particularly important. After stroke, the activation amplitude of peroneus longus is significantly reduced and loses characteristic correlated activation with other ankle muscles [42], indicating loss of corticospinal tract innervation and spinal interneuron regulation. Although the reticulospinal tract may compensate for corticospinal loss [43], this compensation is limited to gross motor control and synergistic patterns [44], whereas ankle eversion requires fractionated, independent muscle control that depends on corticospinal integrity [45]. The peroneus longus may therefore be inadequately reinnervated by available compensatory pathways, potentially contributing to its pronounced size reduction. However, this interpretation remains tentative, as our ultrasound data cannot confirm neural mechanisms.

FHB thickness reduction was another significant finding in our stroke cohort (*d* = 0.76). This selective vulnerability can be understood from anatomical, functional, and neurophysiological perspectives. Anatomically, FHB contributes to medial longitudinal arch stability during static balance by generating a moment opposing dorsiflexion at the MTP joint. Its active contraction pre‐tensions the plantar fascia, enhancing passive arch support and increasing forefoot stiffness [46]. This continuous weight‐bearing demand during standing and gait makes FHB particularly vulnerable to disuse when post‐stroke mobility is reduced—a mechanism that may contribute to the pronounced thickness reduction observed in our patients.

Functionally, FHB plays a critical role in fine motor control for balance maintenance. In healthy controls, FHB thickness showed strong negative correlations with mediolateral displacement under eyes‐closed conditions (*r* = −0.52, *p* < 0.001), indicating that greater FHB size is associated with more stable balance when visual input is withdrawn and plantar proprioception becomes essential. This functional reliance on FHB for postural stability may be achieved through two complementary neural mechanisms. At the reflex level, it depends on precise CNS modulation of long‐latency stretch reflexes through cortical motor networks, with reflex gains dynamically adjusted by descending signals to integrate high‐order motor commands, continuously fine‐tuning muscle tension to counteract minor perturbations [47]. At the motor unit level, FHB enables flexible motor unit recruitment and precise force modulation through frequency adjustments of low‐threshold units under CNS control, avoiding reduced flexibility from excessive forefoot stiffness [48]. Both reflex‐level and motor‐unit‐level regulation depend on low innervation ratios to support fine, graded force control [49]. Stroke significantly impairs voluntary activation of affected muscles [50]. Muscles that depend on such dense cortical input and finely graded control, of which FHB is a prime example [48, 51, 52], are therefore especially vulnerable when this drive is compromised. This heightened sensitivity to central drive loss helps explain why FHB, among all intrinsic foot muscles, showed the most pronounced thickness reduction in our stroke cohort.

The pattern of muscle involvement was not uniform across all measured muscles. Among intrinsic muscles, only FHB showed significant thickness reduction, whereas FDB and FDL did not. This is likely attributable to differences in their functional demands: FHB is a key stabilizer of the medial longitudinal arch and is continuously loaded during stance and gait, whereas FDB and FDL primarily act on the lesser toes and contribute less to arch support [53, 54]. Given that weight‐bearing and antigravity muscles are particularly susceptible to size reduction when activity is reduced [55], FHB may be more vulnerable to disuse‐related size reduction because of its greater weight‐bearing demands, whereas FDB and FDL with lower baseline loading may be relatively spared.

Similarly, the absence of significant change in FHL contrasts with the marked reduction in FHB. Although both muscles contribute to toe flexion, FHB is an intrinsic muscle primarily involved in arch support and postural control, whereas FHL is a larger extrinsic muscle that contributes primarily to gross motor output during push‐off [53, 54]. Intrinsic foot muscles such as FHB have been shown to possess substantially fewer motor units than other human muscles [51], and they exhibit the strongest motor unit synchrony—a marker of dense cortical input—among all muscles examined [52]. In addition, extrinsic foot muscles such as FHL may be relatively preserved through compensatory activation when intrinsic muscle function is compromised [56]. However, whether this pattern persists over time remains to be established in longitudinal investigations.

Beyond between‐muscle differences, thickness and CSA changes within the same muscle were not always consistent. The inconsistency may be attributable to the different nature of these measurements: thickness is a linear measurement along a single axis, whereas CSA requires tracing the entire muscle boundary. CSA measurements have greater inherent variability than thickness measurements [31], which may have reduced the statistical power to detect CSA differences in small, deeply located muscles such as FDB and FHL. Biologically, however, thickness and CSA capture fundamentally different aspects of muscle structure: linear dimensions that thickness reflects along the muscle fibers are influenced by sarcomere length, fascicle architecture, and passive tension [57, 58, 59], whereas CSA reflects the cross‐sectional profile, influenced by fiber cross‐sectional area and the number of parallel sarcomeres [60, 61]. These two dimensions can be differentially affected by stroke‐induced changes, which may explain why thickness differences were detected in some muscles while CSA differences were not [62]. Notably, no intrinsic muscle showed significant differences in both thickness and CSA, whereas both extrinsic muscles (PL and TA) did—a pattern suggesting that extrinsic muscles may be more sensitive indicators of post‐stroke changes within the first 12 months, whereas intrinsic muscles may be relatively spared during this period. Extrinsic muscles such as PL and TA bear high mechanical demands during gait and serve antigravity functions; reduced ambulatory activity may thus lead to more pronounced size reduction, consistent with the known response of antigravity and postural muscles to disuse [55]. In contrast, intrinsic foot muscles are activated during standing to support the medial longitudinal arch [53, 63]. This standing‐related activation may persist even in patients with limited mobility, potentially providing relative protection. However, no published study has directly compared the time course of intrinsic versus extrinsic foot muscle changes within the first year post‐stroke, and these questions require longitudinal investigation.

In addition to the differential patterns observed in muscles, plantar fascia also showed significant structural changes. The significant increase in plantar fascia thickness in the middle and posterior segments may represent a compensatory response to altered biomechanical loading patterns. This structural adaptation could reflect the body’s attempt to maintain foot stability in the presence of compromised muscular control. In addition, the observed plantar fascia thickening may also be explained, at least in part, by vascular mechanisms. Ischemic stroke can trigger a systemic vascular inflammatory response–peripheral endothelial activation and upregulation of adhesion molecules, which can persist for months [64] and promote atheroprogression in peripheral arteries [65]. Such vascular compromise may lead to chronic microvascular insufficiency in lower extremity tissues, and chronic ischemia has been associated with connective tissue remodeling and fibrosis [66]. This mechanism may be particularly relevant to our cohort: 80% of patients had ischemic stroke, and the mean time since stroke was 6.8 months, a period during which inflammatory responses and subsequent tissue remodeling can persist. Thus, plantar fascia thickening may reflect, in part, vascular insufficiency rather than biomechanical factors alone. This interpretation, however, remains to be confirmed.

Comparison of our findings with previous studies reveals a complex picture. Consistent with Calvo‐Lobo et al. [30], we found no significant group differences in FHB CSA, nor in FDB or FHL thickness. Opposite directions were noted for FHB thickness and plantar fascia: reduced FHB thickness with increased middle and posterior plantar fascia thickness in the present stroke cohort (3–12 months post‐stroke), versus increased FHB thickness with decreased middle and anterior plantar fascia thickness in the chronic stroke cohort of Calvo‐Lobo et al. [30].

The FHB discrepancy may reflect differences in disease stage and the nature of the thickness measurement. Thickness reflects linear dimensions along the muscle fibers [62]. Our cohort was in the subacute to early chronic phase (3–12 months post‐stroke), during which reduced neural drive and disuse can lead to muscle flaccidity and decreased resting tone [67]. This could alter the overall foot posture and may manifest as reduced thickness. Calvo‐Lobo et al. studied chronic patients (> 12 months). Over time, flexor spasticity and abnormal plantarflexion posture may shorten the FHB, and prolonged muscle shortening has been shown to be associated with serial sarcomere loss [59], which may paradoxically increase muscle thickness. Notably, FHB thickening in chronic stroke may not represent functional recovery. Larger FHB muscles have been associated with poorer balance performance in healthy individuals [68], and excessive foot pronation correlates with increased FHB CSA and decreased innervation density, impairing fine CNS regulation [69, 70].

Measurement site and disease stage may also account for the plantar fascia discrepancy. The posterior and middle fascia primarily support the medial longitudinal arch during stance, whereas the anterior portion transmits forces during push‐off via the windlass mechanism [71]. In our early‐stage cohort, increased middle and posterior fascia thickness likely reflects a compensatory response to intrinsic muscle weakness [72]. In the chronic cohort, decreased middle and anterior fascia thickness may reflect disuse and altered loading—prolonged abnormal gait with reduced push‐off forces may lead to insufficient tensile loading and consequent thinning [71]. Our study further extends the scope to extrinsic muscles (e.g., peroneus longus), which were not examined in previous studies.

The structural‐functional dissociation identified in this study has important clinical implications. Although post‐stroke balance impairment is clearly multifactorial— involving altered neural pathways, impaired sensory integration, task‐specific biomechanical demands, and cognitive factors such as attention and executive function [73]—our findings highlight that peripheral muscle structure alone cannot explain balance performance. The absence of structure‐function correlations in stroke patients suggests that the primary deficit lies not in muscle size per se, but in the centrally mediated control of these muscles. Therefore, rehabilitation interventions should prioritize neuromuscular control training—aimed at restoring central drive and improving motor output—rather than focusing solely on muscle strengthening. The multivariable regression model for healthy controls, explaining 38% of balance variance through FHB and peroneus longus thickness, provides insight into the normal structure‐function relationship. This baseline understanding can guide rehabilitation strategies by identifying which muscles are most critical for balance control under normal circumstances, even though these relationships are disrupted post‐stroke.

Several limitations should be acknowledged. First, the cross‐sectional design precludes causal inference regarding the temporal relationship between structural changes and functional outcomes. Second, the static balance assessment may lack the sensitivity to fully capture the dynamic role of plantar muscles; future studies should include dynamic or perturbed balance tasks. Third, heterogeneity in stroke lesion location and severity may influence the observed relationships, though subgroup analyses suggested minimal effects of stroke type, disease stage, or hemiplegic side. Fourth, formal intra‐operator reliability testing for ultrasound measurements was not performed, and ultrasound is inherently operator‐dependent. Fifth, quantitative B‐mode echogenicity analysis was not performed, limiting our ability to distinguish true tissue atrophy from other structural changes. Sixth, only a limited set of foot muscles was selected, and trunk stabilizers were not evaluated; their potential contribution to the observed dissociation therefore remains unknown. Seventh, formal test‐retest reliability for our CoP protocol was not conducted, despite known variability of CoP measures in stroke populations. Finally, the absence of electromyographic data prevents direct confirmation of muscle activation deficits.

This study demonstrates that stroke patients exhibit significant foot structural alterations characterized by selective muscle size reduction and plantar fascia thickening. However, the strong structure‐function correlations observed in healthy controls are absent post‐stroke, indicating structural‐functional dissociation. These findings suggest that post‐stroke balance dysfunction cannot be explained by peripheral structural changes alone and is likely dominated by central nervous system impairments—a conclusion that must be tempered by the possibility that the static standing task was insufficiently demanding to reveal the functional role of plantar structures. Clinical rehabilitation should therefore emphasize neuromuscular control training and neural pathway restoration rather than focusing solely on muscle strengthening. Future longitudinal studies are needed to track the evolution of structure‐function relationships during stroke recovery and evaluate the effectiveness of targeted neurorehabilitation interventions.

## Author Contributions

**Xiaoya Zhang:** writing – original draft, software, data curation, writing – review and editing, visualization, supervision. **Yaobin Wu:** validation, formal analysis, data curation. **Guirong Liu:** conceptualization, validation, investigation.

## Funding

The authors have nothing to report.

## Ethics Statement

This study was approved by the Ethics Committee of Third Affiliated Hospital of Sun Yat‐Sen University.

## Consent

The study was conducted according to the Declaration of Helsinki. All participants provided written informed consent.

## Conflicts of Interest

The authors declare no conflicts of interest.

## Supporting information


**Figure S1:** Percentage changes in foot structures in stroke patients relative to controls.

## Data Availability

The data that support the findings of this study are available from the corresponding author upon reasonable request.
