# Supplementary material for: Foot Muscle Size and Balance in Stroke: Evidence of Structural‐Functional Dissociation
Source: J Foot Ankle Res. 2026 Aug 31;19(3):e70206. doi: 10.1002/jfa2.70206 (PMC13530402; doi:10.1002/jfa2.70206)
Supplement: Supplementary file 1 [file JFA2-19-e70206-s001.docx]

Supplementary figure S1. Percentage changes in foot structures in stroke patients relative to controls.

Radar chart showing relative changes in muscle thickness and cross-sectional area. Positive values indicate increases; negative values indicate reductions in stroke patients.
